# Influence of Magnetic Sublattice Ordering on Skyrmion Bubble Stability in 2D Magnet Fe_5_GeTe_2_

**DOI:** 10.1021/acsnano.4c00853

**Published:** 2024-07-08

**Authors:** Max T. Birch, Fehmi S. Yasin, Kai Litzius, Lukas Powalla, Sebastian Wintz, Frank Schulz, Alexander E. Kossak, Markus Weigand, Tanja Scholz, Bettina V. Lotsch, Gisela Schütz, Xiuzhen Z. Yu, Marko Burghard

**Affiliations:** † Max Planck Institute for Intelligent Systems, Heisenbergstraße 3, Stuttgart 70569, Germany; ‡ RIKEN Center for Emergent Matter Science, Hirosawa 2-1, Wako 351-0198, Japan; § Center for Nanophase Materials Sciences, Oak Ridge National Laboratory, Oak Ridge, Tennessee 37830, United States; ∥ Max Planck Institute for Solid State Research, Heisenbergstraße 1, Stuttgart 70569, Germany; ⊥ Helmholtz-Zentrum Berlin für Materialien und Energie GmbH, Hahn-Meitner-Platz 1, Berlin 14109, Germany; # Department of Materials Science and Engineering, Massachusetts Institute of Technology, Cambridge, Massachusetts 02139, United States; ¶ University of Munich (LMU), Butenandtstraße 5-13 (Haus D), München 81377, Germany

**Keywords:** skyrmions, bubbles, 2D magnets, Fe_5_GeTe_2_, microscopy

## Abstract

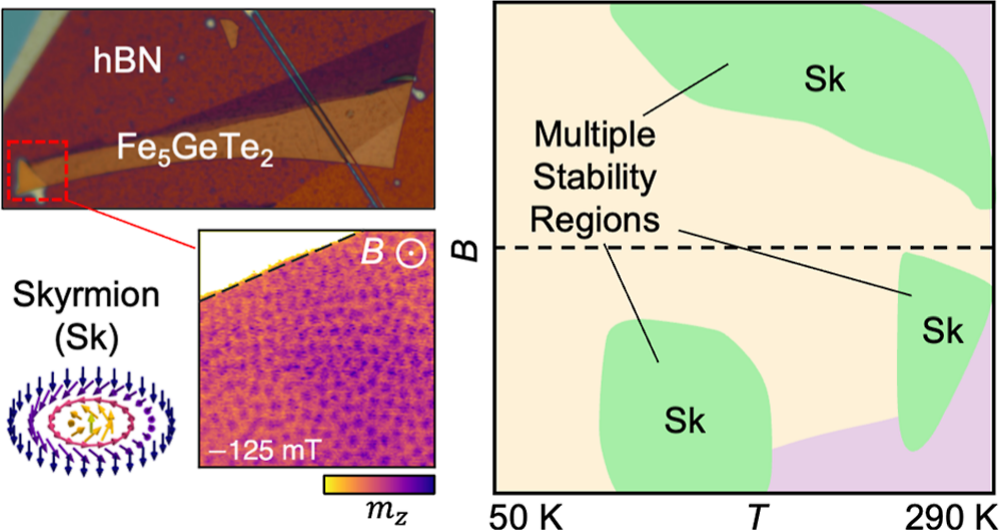

The realization of above room-temperature ferromagnetism in the two-dimensional (2D) magnet Fe_5_GeTe_2_ represents a major advance for the use of van der Waals (vdW) materials in practical spintronic applications. In particular, observations of magnetic skyrmions and related states within exfoliated flakes of this material provide a pathway to the fine-tuning of topological spin textures via 2D material heterostructure engineering. However, there are conflicting reports as to the nature of the magnetic structures in Fe_5_GeTe_2_. The matter is further complicated by the study of two types of Fe_5_GeTe_2_ crystals with markedly different structural and magnetic properties, distinguished by their specific fabrication procedure: whether they are slowly cooled or rapidly quenched from the growth temperature. In this work, we combine X-ray and electron microscopy to observe the formation of magnetic stripe domains, skyrmion-like type-I, and topologically trivial type-II bubbles, within exfoliated flakes of Fe_5_GeTe_2_. The results reveal the influence of the magnetic ordering of the Fe1 sublattice below 150 K, which dramatically alters the magnetocrystalline anisotropy and leads to a complex magnetic phase diagram and a sudden change of the stability of the magnetic textures. In addition, we highlight the significant differences in the magnetic structures intrinsic to slow-cooled and quenched Fe_5_GeTe_2_ flakes.

Since the observation of magnetic ordering in CrI_3_ and Cr_2_Ge_2_Te_6_,^1,2^ two-dimensional (2D) van der Waals (vdW) magnets have seen widespread research interest. Due to their well-defined atomically flat layers, which typically lack dangling bonds, they are well-suited for being stacked into heterostructures and combined with the growing catalogue of other 2D materials.^3^ This enables exploitation of all manner of interfacial and proximity effects, which can be expected to give rise to a rich variety of physical phenomena with potentially wide-ranging technological applications.^4^ In particular, the discovery of 2D magnets offers a diverse material platform for spintronics, which aims to utilize the spin degree of freedom of electrons in storage and logic devices.^5^ Along these lines, a number of prototype spintronic devices have already been demonstrated.^6,7^

The envisioned applications depend crucially on the Curie temperature, *T*_C_, of the magnetic material: it should be above room temperature for convenient operation of proposed devices. Most 2D magnets discovered so far have ordering temperatures well below 300 K—for example, CrI_3_ and Cr_2_Ge_2_Te_6_ both have a *T*_C_ around 60 K in bulk, which is reduced in the monolayer limit. A notable exception to this trend is the family of materials Fe_*n*_GeTe_2_ (FGT), where crystal structures with 2.5 < *n* < 5 have been synthesized,^8−10^ and those with *n* = 6, 7 have been proposed.^11^ The most widely studied of these materials is Fe_3–*x*_GeTe_2_ (F3GT, where *x* indicates some Fe deficiency), which initially stood out for having a *T*_C_ of up to 230 K.^12−14^ However, recently, *T*_C_ values approaching room temperature have been demonstrated for Fe_4–*x*_GeTe_2_ (F4GT, 270 K)^15^ and Fe_5–*x*_GeTe_2_ (F5GT, 310–330 K).^16^ For all FGT materials, this *T*_C_ is tunable by both chemical substitution^17−19^ or electrostatic and ionic gating.^20,21^

One potential avenue of spintronics application research involves the exploitation of topological spin textures, such as magnetic skyrmions, for racetrack memory or neuromorphic devices.^22,23^ Magnetic skyrmions, nanoscale quasi-particles with a unitary topological charge,^24,25^ have been found in a range of magnetic materials and are typically stabilized by either the Dzyaloshinskii–Moriya interaction (DMI) or dipolar interaction.^26,27^ Here, it is crucial to draw attention to the distinction between magnetic skyrmions in comparison to magnetic bubbles,^27^ which remains a point of contention within the wider literature. For the purposes of this paper, we will define skyrmions as magnetic objects with topological charge *Q* = 1, which exhibit a single chirality throughout the host material, typically due to the presence of DMI. On the other hand, magnetic bubbles are stabilized by the dipolar interaction and come in two flavors: type-I bubbles (often called skyrmionic bubbles), which possess the same *Q* = 1 topology as skyrmions but are typically found with both left- and right-handed chirality within a single sample; and type-II bubbles, which have a *Q* = 0 and are composed of two domain walls joined by a pair of Bloch lines.

In the case of van der Waals magnets, magnetic skyrmions or bubbles have now been observed by microscopy in a range of systems,^28,29^ and in particular in F3GT.^30−36^ Recently, investigations have extended to F5GT, with various reports of real-space spin textures in thin flake or lamella samples.^37,38^ There are claims that there may be some form of DMI present in F5GT due to supercells within the crystal structure breaking inversion symmetry.^39,40^ On the other hand, there exist several reports of F5GT exhibiting skyrmionic type-I bubbles of both chiralities and type-II bubbles, which indicates, at best, a weak DMI.^40,41^ Recent studies of few-layer F5GT flakes appear to agree with this apparent lack of significant DMI, showing Bloch-type stripe and bubble states with a strong layer number dependence.^42,43^ However, there are reports showing DMI-induced Néel-type skyrmions in the partially substituted (Fe_0.5_Co_0.5_)_5_GeTe_2_, attributed to a noncentrosymmetric structure.^44−46^ The matter is further complicated by the study of two types of F5GT—defined by whether the single crystals are quenched or slow-cooled after their high-temperature growth, which appear to show significantly different structural and magnetic properties.^9,47^ Therefore, we saw a need for a comprehensive study investigating the potential formation and stability of skyrmions and related real-space spin textures in F5GT.

In this work, we investigate the spin textures hosted in exfoliated flakes of F5GT prepared from single crystals of both the slow-cooled and quenched varieties. Using a combination of magnetometry, scanning transmission X-ray microscopy (STXM), and Lorentz transmission electron microscopy (LTEM), in the slow-cooled type F5GT, we observe the formation of Bloch-type stripe domains, alongside type-I bubbles with *Q* = 1 and type-II bubbles *Q* = 0. We demonstrate a significant alteration of the stability of these spin textures below 150 K, which appears to coincide with a change in the uniaxial anisotropy due to the ordering of the Fe1 sublattice around this temperature. Furthermore, we show that the size and shape of the flake sample have a considerable effect on the spin texture formation due to the delicate balance of magnetic energy terms. Finally, we highlight the significant differences in the spin textures observed in the slow-cooled and quenched F5GT flakes.

## Results

### Two Types of Fe_5_GeTe_2_ Crystals

**Figure 1:**
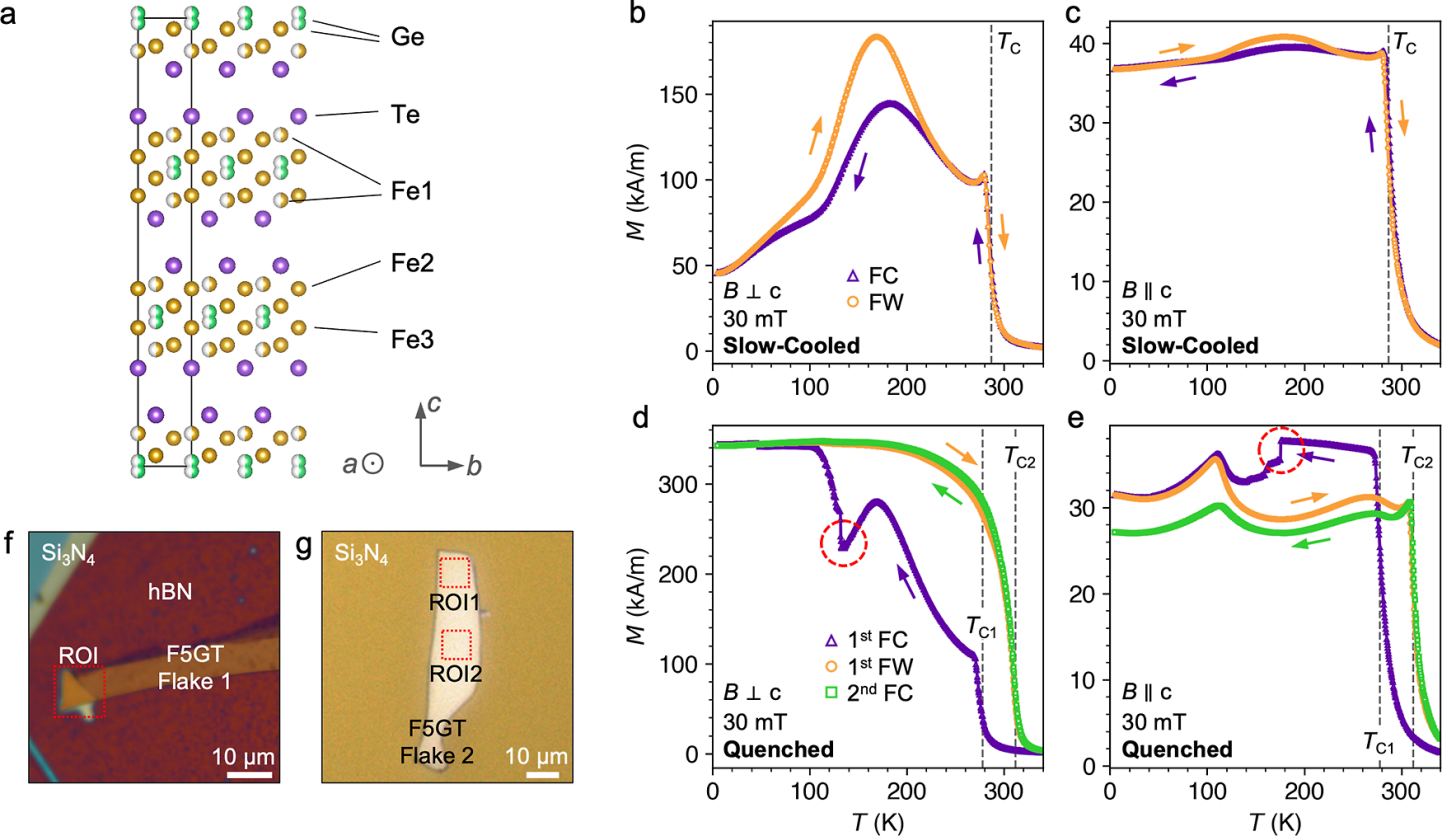
Structure and characterization of the slow-cooled and quenched Fe_5_GeTe_2_ samples. (a) Crystal structure of Fe_5_GeTe_2_, with the positions of Fe1, Fe2, Fe3, Ge, and Te indicated. The half-colored Fe1 and Ge atoms indicate split vacancy sites. (b–e) Magnetization *M* of the bulk slow-cooled (b,c) and the quenched (d,e) Fe_5_GeTe_2_ crystals. Measurements were performed as a function of temperature *T* following both field-cooling (FC) and field-warming (FW) processes, under a field of 30 mT applied either perpendicular (*B*⊥*c*) or parallel (*B*∥*c*) to the *c* crystal axis. (f,g) Optical images of the two investigated slow-cooled flakes, stamped on Si_3_N_4_ membranes. Flake 1 was capped with an hBN flake and investigated in the STXM measurements. Flake 2 was investigated in the LTEM measurements. The labeled regions of interest (ROI, red dashed lines) indicate the areas investigated in the imaging experiments. The ROI of flake 1 has a thickness of ∼120 nm, while flake 2 has a thickness of ∼150 nm.

The crystal structure of F5GT is shown in Figure 1a, revealing the position of three different Fe species within the lattice: Fe1, Fe2, and Fe3. Previous X-ray diffraction and scanning transmission electron microscopy studies have shown that the Fe2 and Fe3 sites have a fixed position, while the Fe1 site is only partially occupied and the Ge site is located on a split position, with the split site occupation depending on the location of Fe1.^16^ Accordingly, the in-plane crystal lattice can exhibit interesting supercell structures, characterized by different local ordering of the Fe1 and Ge lattice sites.^39^ These local orderings are thought to break inversion symmetry—while the average crystal structure with rhombohedral space group *R*3̅*m* is centrosymmetric, the locally ordered regions could possess the noncentrosymmetric space group *R*3*m*.^41^ Similar to the arguments concerning local vacancies breaking symmetry in F3GT,^35^ this broken inversion symmetry in F5GT may result in a local DMI and thus DMI-stabilized helimagnetic spin textures.^39^

The crystal structure of F5GT is significantly altered depending on the cooling method utilized after the growth procedure. Bulk F5GT crystals are typically characterized by numerous stacking faults, manifesting in elongated diffraction peaks (streaking) along the *c* axis.^47^ However, this disorder is absent in crystals rapidly quenched from high temperature.^16^ This quenching is thought to stabilize a more ordered structural phase which exists above 550 K, resulting in a metastable structure at room temperature. The magnetic properties of crystals fabricated by the two processes have also been reported to be quite different.^9^ To explore this, we synthesized bulk F5GT crystals of both varieties (see Materials and Methods).

Magnetometry measurements were first performed on the synthesized bulk crystals (see Materials and Methods). The magnetization *M*(*T*) of the slow-cooled bulk F5GT crystal is plotted as a function of temperature in Figure 1b,c, with a 30 mT magnetic field applied either perpendicular or parallel to the *c* axis, respectively, for both field-cooling (FC) and field-warming (FW) processes. The data reveals that the sample has a *T*_C_ of 286 K. Figure 1b demonstrates the complex behavior of the crystal for fields applied in-plane (perpendicular to the *c* axis), where *M*(*T*) initially increases with decreasing *T*, up to maximum value at approximately 160 K, and displays a further cusp at around 110 K. In contrast, for the out-of-plane applied field (parallel to the *c* axis), *M*(*T*) is mostly flat below *T*_C_, as shown in Figure 1c. Previous studies have linked this temperature-dependent *M* to the behavior of the moments on the partially occupied Fe1 sites, which only order at lower temperatures (discussed in more detail later).^9^ It has been argued that the decrease in *M* at low temperatures could be evidence for canting of the moments or even ferrimagnetic ordering.^48^

The *M*(*T*) behavior of the quenched sample is considerably different, as shown in Figure 1d,e. Looking at the first FC data in Figure 1d, *M*(*T*) initially behaves similarly to the slow-cooled sample, ordering at *T*_C_ of ∼276 K and showing a steady increase up to a maximum at ∼180 K. However, below 140 K, there is a sudden and dramatic change in *M*(*T*) (marked by the dashed red circle). A similar transition is seen beginning at 175 K in the out-of-plane data in Figure 1e (separate crystals were used for each field orientation to reproduce the irreversible behavior). Upon finishing the FC process, and performing further FW and FC protocols, the subsequent data shows that the magnetic behavior of the sample has irreversibly changed: the sample no longer displays the complex *M* behavior for in-plane fields and exhibits a much higher *T*_C_ of ∼310 K. Previous work has linked this sudden change to a magneto-structural transition in the quenched crystal: the ordering of the Fe1 moments appears to be linked to a reappearance of the stacking faults within the crystal structure, presumably due to some magnetoelastic behavior.^16^ The significant differences in the bulk magnetic behavior naturally raise questions over the nature of the spin textures hosted in exfoliated flakes of each of these crystals.

For the subsequent imaging experiments, flakes of F5GT were stamped onto Si_3_N_4_ membranes. In the case of X-ray microscopy, both slow-cooled and quenched F5GT flakes were capped with an exfoliated flake of hexagonal boron nitride (hBN) to prevent further oxidation (see Materials and Methods). For the LTEM measurements, slow-cooled material was utilized, and the flakes were left uncapped to improve the electron transmission through the stack. In the following, we present microscopy data of both types of F5GT flakes. However, contrary to expectations in the literature, we observed significant structural defects and disordered magnetic textures in the quenched flakes, which made their thorough investigation challenging. Thus, we first primarily focus on the well-characterized and reproducible behavior observed in the slow-cooled flake samples and return to the quenched F5GT at the end of the article. Optical micrographs of the two investigated slow-cooled flakes (denoted flake 1 and flake 2) are shown in Figure 1f,g where the region of interest (ROI) examined in subsequent STXM and LTEM images is highlighted.

### Microscopy of Slow-Cooled Flakes

STXM acquires magnetic contrast images by measuring the X-ray transmission through the sample and exploiting the effects of X-ray magnetic circular dichroism (XMCD), yielding a signal scaling with the out-of-plane magnetization, *m*_*z*_ (see Figure S1 and Materials and Methods). In contrast, LTEM exploits the Lorentz force induced by the local in-plane magnetic fields produced by the sample magnetization to achieve magnetic contrast (see Materials and Methods). The techniques therefore give complementary information, and the in-plane field sensitivity allows LTEM to determine the domain wall type (Bloch or Néel) and therefore distinguish the nature of the spin textures in the sample.

**Figure 2:**
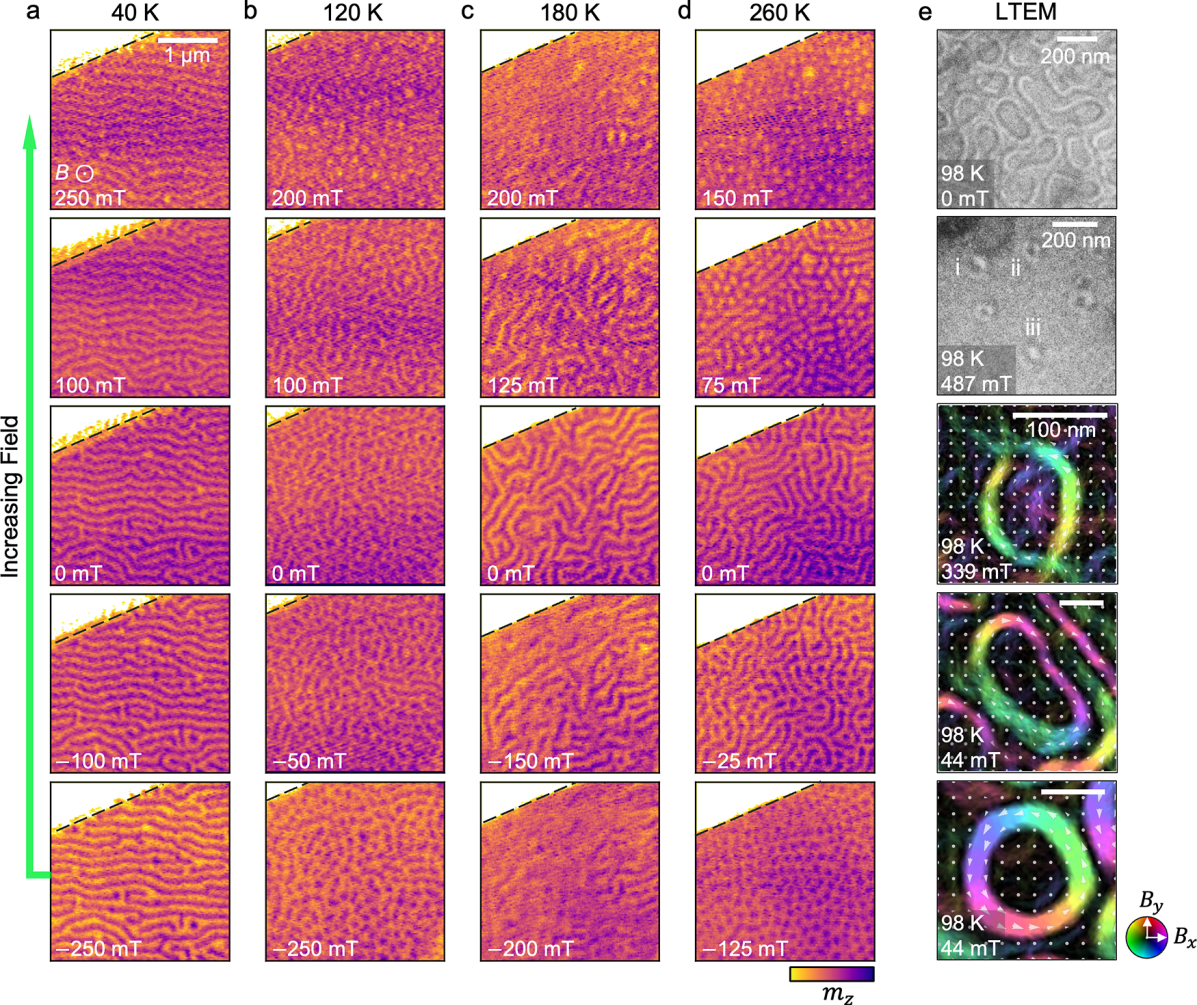
X-ray and LTEM imaging of slow-cooled Fe_5_GeTe_2_ under an out-of-plane applied magnetic field. (a–d) X-ray micrographs of the ROI of the exfoliated slow-cooled Fe_5_GeTe_2_ flake 1, acquired at a range of temperatures and applied out-of-plane magnetic fields. The images were taken following the field-sweep protocol: at each temperature, the out-of-plane applied magnetic field was increased stepwise after being initialized at −250 mT. The color map scales with the out-of-plane magnetization of the flake, *m*_*z*_ (light is down, dark is up). (e) Selected LTEM images of the slow-cooled Fe_5_GeTe_2_ flake 2 acquired at 98 K. The upper two panels show the raw contrast achieved when defocusing the electron beam, revealing magnetic contrast of stripe domains and, at higher applied fields, magnetic bubble states. The lower three panels show the in-plane magnetic induction reconstructed using the transport-of-intensity equation (TIE) of the different observed bubble objects: type-II bubbles and type-I bubbles of left- and right-handed chirality. The color wheel indicates the direction of the in-plane magnetic induction.

To investigate the stability of spin textures within flakes of the slow-cooled sample, we first performed STXM imaging of the ROI of flake 1. Images were acquired as a function of increasing out-of-plane applied magnetic field, starting from −250 mT [which we call the field sweep (FS) procedure]. The results for a range of temperatures are shown in Figure 2a–d, revealing the formation of stripe and skyrmion states. We defined the *T*_C_ of the flake sample as the temperature at which we could no longer observe stripe domain contrast at 0 mT, which was found to be ∼275 K, similar to that seen in the bulk. Close to *T*_C_, the flake exhibits the formation of dense arrays of bubbles at both negative and positive applied fields, with a stripe state forming around 0 mT, as shown in the 260 K data in Figure 2d. At slightly lower temperatures, such as the 180 K data in Figure 2c, the sample primarily exhibits stripe domains, with only a few isolated bubbles forming at high positive applied fields.

**Figure 3:**
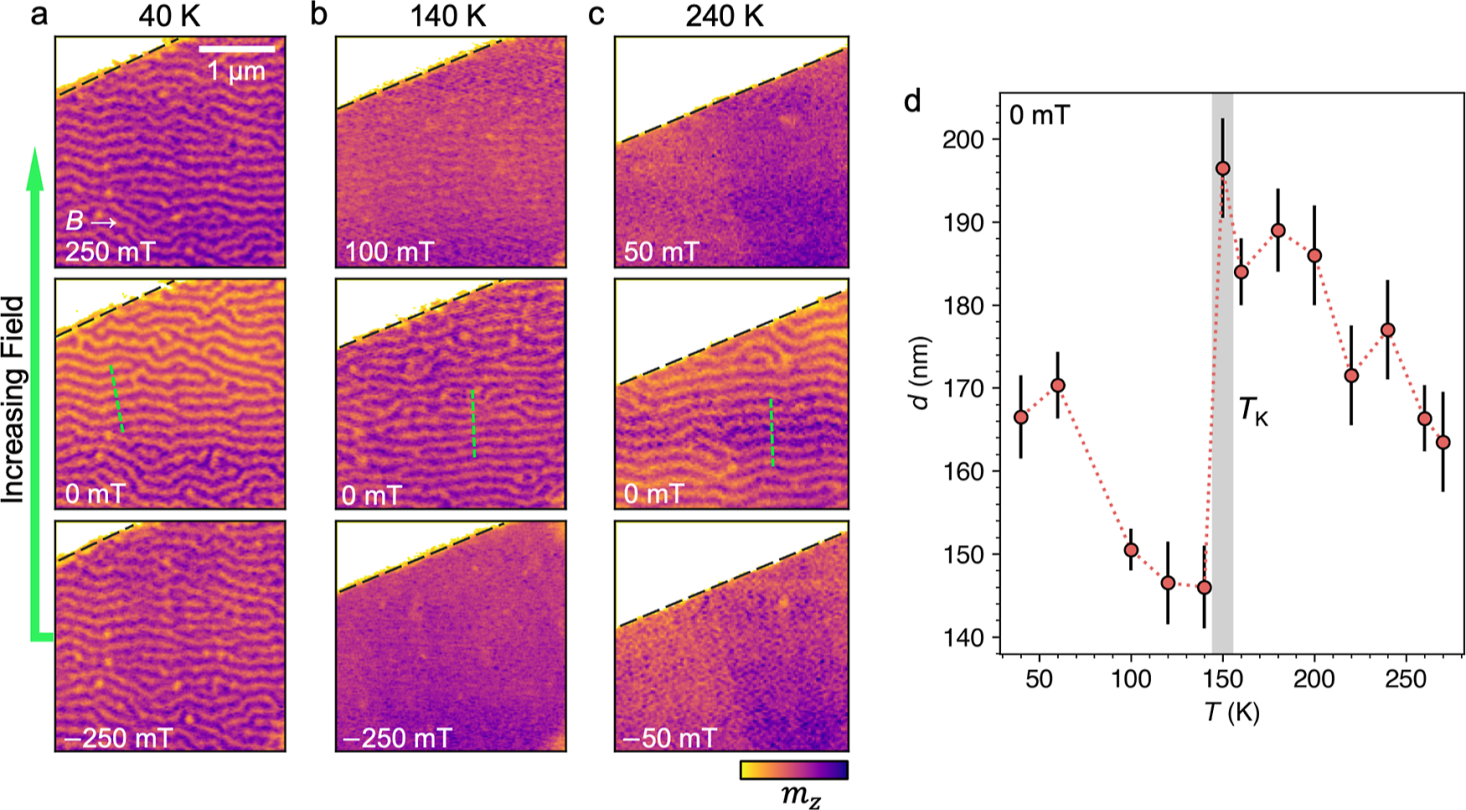
X-ray imaging of slow-cooled Fe_5_GeTe_2_ under an in-plane applied magnetic field. (a–c) X-ray micrographs of the ROI of the exfoliated Fe_5_GeTe_2_ flake 1, acquired at a range of temperatures and applied in-plane magnetic fields. The images were taken following the field-sweep protocol: at each temperature, the in-plane applied magnetic field was increased stepwise after being initialized at −250 mT. The color map is scaled with the out-of-plane magnetization of the flake, *m*_*z*_ (light is down, dark is up). Dashed green lines indicate example locations of the linescans used to evaluate the stripe domain spacing. (d) Average stripe domain size *d* at 0 mT as a function of the sample temperature. Error bars indicate the standard error acquired when averaging the results of multiple line scans across the domains in each image. *T*_K_ indicates the temperature at which the domain size suddenly changes, which we argue is due to a sudden alteration of the uniaxial anisotropy by the Fe1 sublattice ordering.

However, below 160 K, we observe the onset of a second temperature range of bubble formation, where once again dense arrays of bubbles are nucleated at both negative and positive applied fields, such as in the 120 K data in Figure 3b. Finally, at yet lower temperatures below 100 K, once again, only the formation of stripe domains is observed, as shown in the 40 K data set in Figure 3a. Taken together, the low-temperature behavior of the F5GT flake therefore contrasts starkly with the monodomain switching seen at low temperatures in F3GT.^36^ Moreover, the formation of two thermally separated regimes of bubble formation is different from most other skyrmion or bubble-hosting materials, with only one exception,^49^ and thus, the interactions responsible for this behavior deserve attention. We shall return to this point in the next section.

To determine the in-plane structure of the observed magnetic states, we performed LTEM imaging of slow-cooled flake 2, with a summary of the results shown in Figure 2e (further LTEM results shown in Figures S2–S5). The first panel reveals the formation of Bloch-type stripe domains at 0 mT, while the second panel shows the simultaneous formation of three types of bubble states realized at higher applied magnetic fields—a type-II bubble (labeled i) and both left- and right-handed chiralities of type-I bubble (labeled ii and iii, respectively). The next three panels show a local map of the in-plane magnetic induction of a type-II bubble, characterized by two Bloch domain walls connected by a pair of Bloch lines, and skyrmion-like type-I bubbles of both left and right chirality, respectively. Throughout the experiments, we predominantly observed type-II bubbles. The presence a mixture of bubble states with different chiralities is strong evidence that the magnetic textures are primarily stabilized by the dipolar interaction, and indicates a lack of significant DMI, which would otherwise favor one chirality of type-I bubble over the other objects.

We performed further X-ray microscopy measurements of the same slow-cooled flake 1, performing the FS procedure while applying an in-plane magnetic field. Figure 3a–c shows a selection of the resulting X-ray micrographs across three temperatures. The F5GT flake only exhibited stripe domains or in-plane uniformly magnetized states for this field configuration. Notably, we observed that the field required to saturate the flake was less than 50 mT at higher temperatures. However, below 160 K, this saturation field rapidly increased, and at 120 K, we were no longer able to saturate the sample in-plane with the 250 mT maximum field available in the X-ray microscope. Another significant result is the characteristic stripe domain size *d* at each temperature, as plotted in Figure 3d. This was determined by acquiring line profiles of the stripe domains parallel to the winding direction of the Bloch walls and defined as the peak-to-peak distance in these line profiles. The results reveal an interesting temperature dependence, where *d* initially increases with decreasing temperature, until there is an abrupt change around 150 K, where there is a dramatic step decrease in *d*, which then continues to increase at lower temperatures. An explanation for this result will be given in the next section.

### Stability of Magnetic Phases

**Figure 4:**
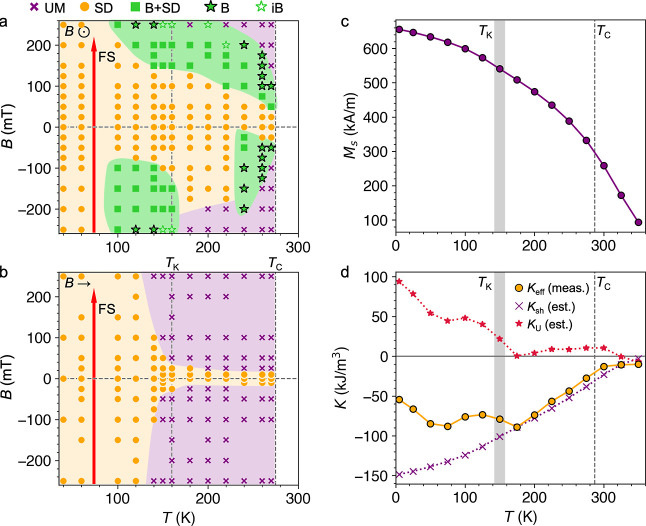
Magnetic phase diagrams of slow-cooled Fe_5_GeTe_2_. (a,b) Magnetic phase diagrams of the slow-cooled Fe_5_GeTe_2_ flake 1, acquired by the field-sweep (FS) process for out-of-plane and in-plane applied magnetic fields, respectively. The presence of the uniformly magnetized (UM, purple crosses), stripe domain (SD, orange circles), dense bubble array (B, green complete stars), isolated bubble (iB, green empty stars), and combined stripe and bubble (B + SD, green squares) states is indicated. Each point corresponds to an acquired X-ray micrograph. The direction of the field sweep, initialized at −250 mT, is indicated by the red arrow. (c) Measured saturation magnetization, *M*_S_, of the bulk slow-cooled F5GT crystal, plotted as a function of temperature. (d) Measured uniaxial anisotropy of the bulk slow-cooled F5GT crystal, *K*_eff_ (orange circles) plotted as a function of temperature, determined from integrating the difference between magnetization loops of the bulk crystal measured with the field parallel and perpendicular to the *c* axis. Also plotted is the estimated shape anisotropy contribution, *K*_sh_ (purple crosses), and the resulting estimated uniaxial anisotropy, *K*_U_ (red stars), obtained from *K*_eff_ – *K*_sh_. In all plots, the value of *T*_C_ and the characteristic transition temperature *T*_K_ is indicated by vertical gray lines.

The behavior seen in the X-ray microscopy data described so far is summarized by the magnetic phase diagrams for both out-of-plane and in-plane applied magnetic fields, plotted in Figure 4a,b, respectively. Different markers are utilized to identify the observed magnetic state in each acquired image. In Figure 4a, the formation of dense skyrmion array states is indicated by the bold star markers, highlighting the two temperature regimes where significant bubble formation is observed. The sudden step change in the saturation field for the in-plane field phase diagram can be seen in Figure 4b. We note that the full phase diagram at low temperature could not be explored due to the upper limit of the applied magnetic field in the X-ray microscope (±250 mT). The field asymmetry of the phase diagrams is due only to the field-sweep direction. For the opposite direction of the field sweep, the phase diagram would appear mirrored in the *x* axis.

As alluded to above, there is one previous example of a material exhibiting thermally separated skyrmion formation regimes: the multiferroic skyrmion host Cu_2_OSeO_3_.^49^ In this material, one thermal fluctuation-induced skyrmion phase is found close to *T*_C_, which is typical for bulk chiral magnet materials. However, the combination of increasing cubic anisotropy and anisotropic exchange also drives skyrmion formation in a second, low-temperature region.^50^ In F5GT, we have observed several interesting behaviors emerging at around 150–160 K, which we label *T*_K_, including pronounced changes in the in-plane saturation field, the characteristic stripe domain size, and the stability of bubble states. Inspired by this intriguing result, we investigated the uniaxial anisotropy in our slow-cooled F5GT sample by performing extensive magnetometry measurements.

In Figure 4c,d, we plot both the saturation magnetization *M*_S_ and the anisotropy of the bulk slow-cooled F5GT crystal as a function of temperature. These values were extracted from magnetization *M* versus applied field magnetometry measurements (see Materials and Methods, Figure S6). By integrating over hysteresis loops obtained with the field applied perpendicular and parallel to the *c* axis, the work done, *W*, to magnetize the sample along each axis can be estimated from^51^
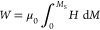
1Thus, the effective anisotropy of the sample can be calculated as *K*_eff_ = *W*_*H*∥*c*_ – *W*_*H*⊥*c*_. The result is plotted by the orange circles in Figure 4d, showing negative values, and a steady decrease with decreasing temperature until about 160 K, where the trend levels out. The measured *K*_eff_ contains the shape anisotropy of the single crystal, which can be expected to be in-plane, hence the negative value of *K*_eff_. Using the experimentally acquired values of *M*_S_, we estimated the contribution of this shape anisotropy following the calculation , which is plotted by the purple crosses in Figure 4d. Note that *N*_d_ is defined by the shape of the sample and was estimated to be 0.57.^49^ From this, the estimated uniaxial anisotropy *K*_U_ of the sample is calculated by subtracting *K*_sh_ from *K*_eff_, with the result also plotted by the red stars in Figure 4d.

While we would stress that this method yields only an estimation of the magnitude of the anisotropy value, the relative values between temperatures are reliable. Thus, the result shows that at higher temperatures, there is likely very little uniaxial anisotropy within the sample. At these temperatures, the flake sample will be dominated by the shape and surface anisotropy contributions. In particular, the observation of out-of-plane domains in the flake indicates that out-of-plane anisotropy remains dominant. However, below the characteristic temperature *T*_K_ of 150 K, there is a sudden change of *K*_U_, corresponding to an increasingly out-of-plane easy-axis. This temperature coincides with the sudden decrease in stripe domain size observed in Figure 3d, the onset of the second region of skyrmion formation at lower *T* shown in Figure 4a, and the observed increase in the IP saturation field below *T*_K_ shown in Figure 4b. While we do not have direct evidence from the current work, based on the previous studies utilizing neutron diffraction and Mössbauer spectroscopy, we suggest that all of these behaviors can be ascribed to the ordering of the FeI sublattice.^16^ In that work, this ordering also seemed to be associated with an increase in the uniaxial anisotropy of the F5GT sample at lower temperatures, as observed here. In the previous work, the sublattice ordering was shown to occur at 100–120 K, while in our sample, these effects are seen at 150 K, which could be due to differences in the stoichiometry, as reflected by the differing *T*_C_ values.

Since the stripe domain states are presumably stabilized by the dipolar interaction, their size should be determined by Kittel’s law.^52^ The increase of uniaxial anisotropy below *T*_K_ would result in larger domains since the domain walls become more energetically costly. However, in Figure 3d, the typical domain size showed a sudden decrease below *T*_K_. Thus, the observed behavior can not only be described by the change in anisotropy, but also we speculate that the sublattice ordering is also altering the saturation magnetization and/or exchange interaction, although the detail requires further study.

**Figure 5:**
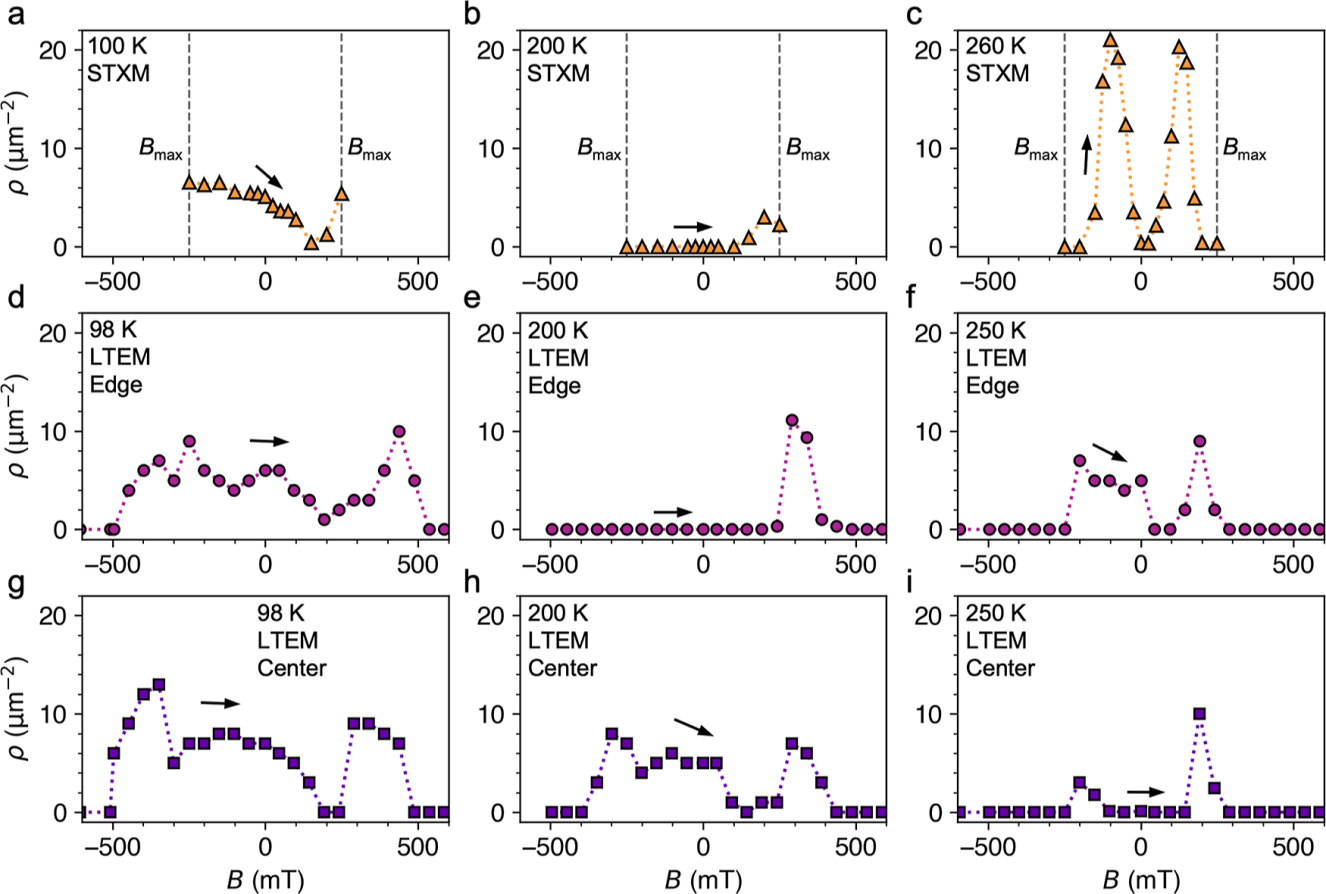
Bubble density in the slow-cooled Fe_5_GeTe_2_ flake 1 sample. (a–i) Bubble density ρ measured as a function of the applied magnetic field using STXM (a–c) and LTEM (d–i) at selected temperatures for the slow-cooled Fe_5_GeTe_2_ flakes. LTEM measurements were performed at both the sample edge (d–f) and center (g–i), as labeled.

Concerning the thermally separated regimes of bubble formation, further details were acquired during the LTEM imaging experiments. In particular, we observed different behavior of the skyrmion stability at the edge and center of the larger flake 2 sample [labeled as ROI1 (edge) and ROI2 (center) in Figure 1g]. This is illustrated in Figure 5, where the observed density of magnetic bubbles for each applied magnetic field at a range of temperatures is plotted for each measurement technique (once again following the FS procedure from negative to positive field). The data in Figure 5a–c, acquired from the STXM data of flake 1, shows the behavior described thus far: at high temperatures close to *T*_C_, a dense array of bubbles forms at both positive and negative applied fields. At 200 K, bubbles are only formed for positive applied fields. However, at temperatures below 150 K, bubble formation is seen across a wide field range. Near identical behavior was observed at similar temperatures at the edge of flake 2, as shown in Figure 5d–f. Note that the higher maximum applied field in the LTEM setup allows us to saturate the sample at all measured temperatures.

In contrast, different behavior is exhibited at the center of the larger flake 2 sample, as shown in Figure 5g–i: skyrmions are stable across most of the field range at all temperatures, which is reminiscent of typical bubble-hosting uniaxial ferromagnets.^53^ This different behavior observed in two regions with the same thickness, but with different proximity to the sample edge, indicates that the direction of the stray field produced by the magnetization may have a dramatic effect on the stability of the magnetic objects within F5GT flakes. Specifically, in the center of the large flake 2, the stray field should be predominantly fixed in the OOP direction. In contrast, at the edge of flake 2, and for the generally smaller flake 1, the stray field will be significantly tilted away from the out-of-plane direction toward the sample boundaries. We speculate that this may suppress the stability of the bubbles for moderate temperatures, presumably by favoring domain walls pointing along the stray-field direction (toward the edge of the sample). However, below 150 K, we suggest that the increased uniaxial anisotropy increases the stability of bubble states in all regions once again. Similar geometry-dependent spin texture stability was observed in LTEM measurements of Fe_1.9_Ni_0.9_Pd_0.2_P.^54^

### X-ray Microscopy of Quenched Flakes

**Figure 6:**
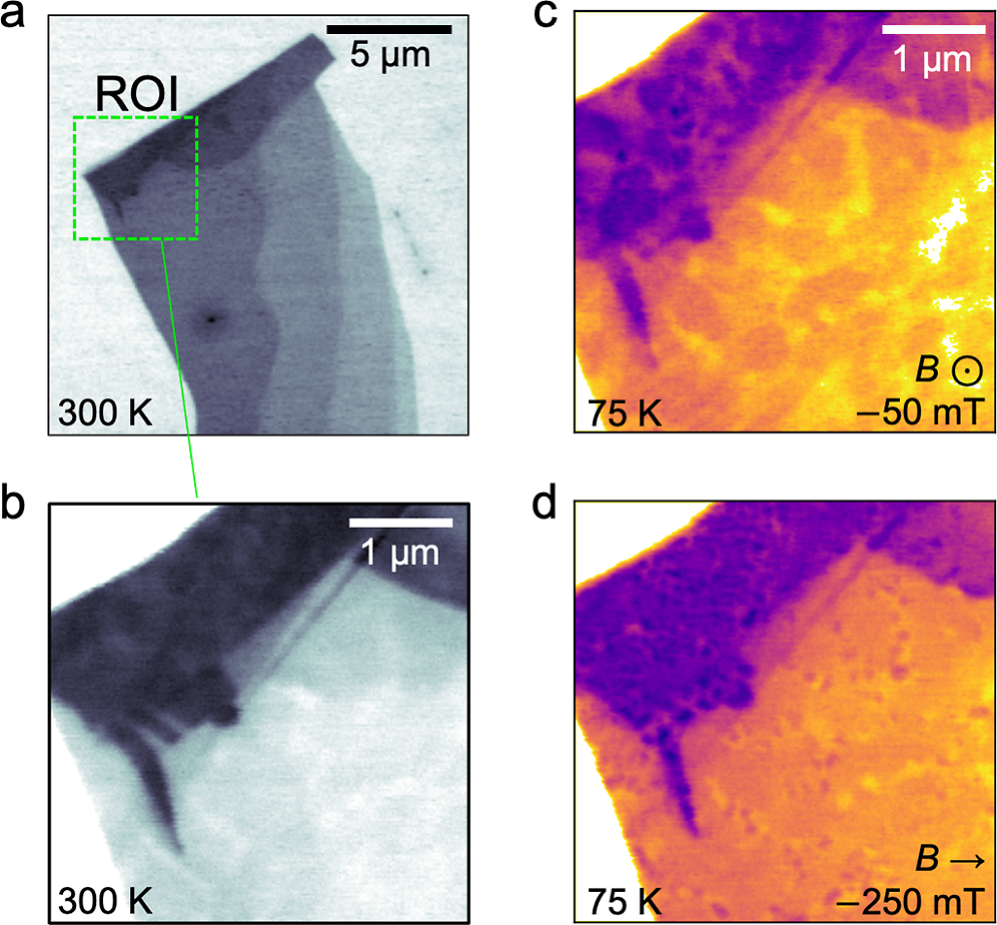
X-ray microscopy of the quenched Fe_5_GeTe_2_ flake. (a) Overview X-ray micrograph of the quenched F5GT flake. The region of interest (ROI) of subsequent images is indicated. (b) X-ray micrograph of the ROI at 300 K before any cooling, revealing significant contrast indicating structural inhomogeneities within the flake sample. (c,d) Two representative X-ray micrographs of a region of the quenched Fe_5_GeTe_2_ flake at 75 K, measured following the field sweep procedure for both out-of-plane and in-plane field, respectively. The images are for a single X-ray polarization, and thus, the contrast is both structural and magnetic in origin, allowing the correlation between magnetic and structural features to be seen.

Before summarizing the results of the slow-cooled samples, we will briefly discuss the STXM results for the quenched flakes. In general, the magnetic states and behaviors of these samples were difficult to characterize, with each flake displaying nonuniform magnetic states. Despite not reaching decisive conclusions, we nevertheless feel it is useful to highlight the significant differences we observed between these samples and the slow-cooled flakes. Some example X-ray micrographs of one quenched flake are shown in Figure 6a,b, measured at 300 K before any cooling procedure—thus, the ferromagnetic was not ordered at this temperature. In Figure 6b, the vein-like regions of light and dark contrast within a single flake thickness therefore indicate structural inhomogeneities, which seemed to exhibit different magnetic behavior to the surrounding material once cooled. We present two example images from field-sweeps performed at 75 K for both out-of-plane and in-plane applied magnetic fields in Figure 6c,d, respectively. In Figure 6c, lighter and darker magnetic domains appear within the lighter structural contrast regions. This more clearly visible for the in-plane field image shown in Figure 6d, where skyrmion/bubble-like objects filled the vein-like regions of lighter structural contrast. To check that these findings were not the result of an unfortunate selection of F5GT flake, we performed measurements on two additional quenched flakes, one of which was from a separately grown bulk crystal. In some cases, we observed regions of more typical stripe states, but in general, the results were inconsistent, and in all three cases showed inhomogeneous spin texture formation and structural inhomogeneities (see Figures S7 and S8).

The image in Figure 6d is reminiscent of a recently published LTEM study of quenched F5GT flakes,^55^ where the authors observed large regions of primarily IP magnetization, together with smaller regions of OOP states hosting skyrmion or Meron states. The large-scale structures appear similar to the vein-like structural contrast features observed in the present data. However, here, we must highlight another study performed on the same quenched F5GT crystals utilized in the present work, which showed more ordered magnetic structures, such as clear stripe and skyrmion states, similar to the slow-cooled flakes studied here.^41^ The difference between the two studies is the sample fabrication method: while we used exfoliation to create F5GT flakes, the previous study utilized focused ion beam milling to fabricate F5GT lamellae. Thus, the different behavior may be due to these processes, where the milling via Ga ions or the straining effect of the exfoliation and transfer method may play a role. In particular, the breakdown of the metastable phase in the quenched samples is associated with the FeI sublattice ordering and magneto-structural transition around *T*_K_. Thus, in our case, any straining of the flake during fabrication may influence these properties and perhaps lead to the inhomogeneous crystal structure within a single flake.

Within the literature, there is a general sentiment that quenched F5GT should display superior crystal quality in comparison to the slow-cooled F5GT due to possessing a sharper X-ray diffraction pattern, with a markedly reduced amount of stacking faults.^9,47^ Thus, it seems many studies have since chosen to focus on quenched F5GT crystals. However, from the perspective of uniform skyrmion and stripe formation, the present data indicates that the slow-cooled F5GT flakes display more reproducible spin texture formation. However, for other experiments and applications, it may be that the properties of the quenched variety are favorable, in particular the increase of *T*_C_ to above room temperature. Taken altogether, it is clear that the details of crystal structure, in particular the presence of defects and stacking faults, play a crucial role in the magnetic behavior of F5GT via the magnetoelastic coupling, and thus we suggest that both varieties of F5GT should be considered for future studies.

## Conclusions

Past studies of F5GT have reported that the Fe1 sublattice magnetically orders at a substantially lower temperature than *T*_C_.^16^ Our measurements reveal the effect that this has on the spin texture formation in slow-cooled F5GT flakes, realized via an increase in the uniaxial magnetocrystalline anisotropy exhibited by the sample. This results in a significantly higher in-plane saturation field, a sudden decrease of the characteristic magnetic domain size, and an increase in the stability of type-I and type-II bubbles at low temperatures, leading to a secondary temperature range of skyrmion bubble formation. Moreover, we have shown that the local geometry of the sample also has a significant impact on the bubble stability, where the bubbles appear more stable toward the center of the large investigated flake. For moderate temperatures, the dipolar-induced stray field energy is large enough to suppress the bubble formation in small flakes and at the edges of larger flakes.

Due to their short exposure to air (∼10 min), the top and bottom few nm of our flakes are likely oxidized, which we have not characterized in the present measurements. However, the Bloch-type domain walls observed in the magnetic textures do not imply the presence of interfacial effects such as DMI, unlike in the existing discussion concerning Néel-type skyrmions in F3GT.^31,32,36^ In addition, we did not see evidence for the short-scale helical ordering observed in a previous work on F5GT^39^—all observed stripe states consisted of larger regions of uniform magnetization separated by narrow Bloch-type domain walls (see Figure S9). The difference may be because the previous study utilized magnetic force microscopy, which is a surface-sensitive imaging technique, as opposed to the transmission techniques applied here.

The combination of strongly temperature-dependent anisotropy and saturation magnetization results in a complex magnetic phase diagram for slow-cooled F5GT, which is significantly different from thin film and bulk skyrmion systems,^56,57^ as well as other 2D magnets.^36^ The delicate balance of the magnetic energy terms should enable tuning of the stability of the hosted spin textures in heterostructure stacks, which we anticipate will be widely explored in future works. Finally, our comparison of the behavior of both slow-cooled and quenched F5GT crystals should encourage further investigations into the details of the defects, atomic vacancies, and layer ordering, with a view toward improving the suitability of F5GT for spintronic applications.

## Materials and Methods

### Sample Fabrication and Characterization

Single crystals of Fe_5_GeTe_2_ were produced in quartz ampules in the presence of iodine as a mineralizer. Powders of the elements were utilized in a ratio of 6:1:2, with the Ge powder placed at the bottom of the ampule and Fe and Te pressed into pellets and placed on top of Ge. After vacuum sealing, the ampules were heated to 750 °C at 120 K/h and were left at this temperature for 2 weeks. For the quenched samples, the ampules were cooled from this high temperature in room-temperature water, while for the slow-cooled samples, the ampules were slowly returned to 21 °C. We performed EDX experiments in a multifunctional TEM (JEOL JEM2800) configured in STEM mode coupled with the NSS spectral imaging system for elemental mapping. This determined the final composition of the slow-cooled samples to be Fe_5.3_GeTe_2.4_ (see Figure S10), while previous results have shown the composition of the quenched samples to be Fe_4.7_GeTe_1.9_.^41^ Magnetometry measurements were performed in a Quantum Design VSM3, with the crystals fixed to a quartz rod sample holder with GE varnish.

Flake samples of both crystal varieties were prepared using an all-dry exfoliation method. First, the FGT bulk crystals were mechanically cleaved and exfoliated onto a polydimethylsiloxane (PDMS) stamp. The PDMS was surveyed using an optical microscope, and flakes with a suitable thickness were selected by their optical contrast. Selected flakes were then stamped onto 100 nm thick Si_3_N_4_ membranes, and each was capped by an exfoliated hexagonal boron nitride (hBN) sheet with thickness on the order of 20 nm. The entire process was performed in ambient conditions, with each side of the flakes exposed to the atmosphere for roughly 15 min. Thus, the first few atomic layers of each flake are expected to be oxidized. The flake thicknesses were measured using atomic force microscopy and electron-filtered transmission electron microscopy, yielding values of 120 and 150 nm for the regions of interest in flakes 1 and 2, respectively.

### Scanning Transmission X-ray Microscopy

X-ray microscopy was performed with the MAXYMUS endstation at the BESSY II electronic storage ring operated by the Helmholtz-Zentrum Berlin für Materialien and Energie. The Si_3_N_4_ membrane chips were mounted to a Cu sample holder and placed inside the microscope chamber. Cooling was achieved by a He flow cyrostat. By controlling the arrangement of four permanent magnets, a magnetic field could be applied in both out-of-plane and in-plane directions, with a maximum magnitude of 250 mT. The X-ray beam was focused to a spot size of approximately 20 nm using a Fresnel zone plate and order-selecting aperture, fixing the maximum spatial resolution. An image was acquired by rastering the sample through the beam spot and measuring the X-ray transmission pixel by pixel using a piezoelectric motor stage. Photons were counted by an avalanche photodiode placed behind the sample. A typical image consisted of a 5 × 5 μm area with a 20 nm pixel size and a dwell time of 1.5 ms, with a total acquisition time of 2–3 min. Magnetic contrast was achieved by exploiting the effects of X-ray magnetic circular dichroism (XMCD) at the Fe L_3_ edge, with a nominal energy of 708.0 eV. The resulting magnetic signal scales with the out-of-plane magnetization, *m*_*z*_ (parallel to the X-ray beam). The presented images of the magnetic domain structure were acquired with a single circular X-ray polarization.

### Lorentz Transmission Electron Microscopy

We performed LTEM experiments in a Thermo Fisher Scientific Talos F200x scanning transmission electron microscope (STEM) using the Gatan 636 TEM liquid nitrogen cryo-TEM holder. LTEM images are the summation of the electron–specimen interaction through the thickness projected onto a 2D detector image plane. We cooled the specimen with zero external magnetic field **B**_ext_ = 0 mT. The objective lens was utilized to apply out-of-plane magnetic fields. When two LTEM images of a magnetic spin texture are acquired at over- and under-defocus values, respectively, the in-plane magnetic inductance of the sample may be reconstructed using the transport-of-intensity equation, assuming a flat electrostatic distribution (thickness).

## Data Availability

The data that support the findings of this study are available from the corresponding author upon request.

## Supporting Information Available

X-ray absorption spectra of the F5GT flakes; additional LTEM images and accompanying phase diagrams; magnetometry data of the bulk slow-cooled F5GT crystal; additional STXM images of two further quenched F5GT flakes; EDX data; and analysis of the LTEM data concerning the helical state at zero applied field (PDF)

## Notes

This manuscript has been authored by UT-Battelle, LLC, under contract DE-AC05-00OR22725 with the US Department of Energy (DOE). The US government retains and the publisher, by accepting the article for publication, acknowledges that the US government retains a nonexclusive, paid-up, irrevocable, worldwide license to publish or reproduce the published form of this manuscript or allow others to do so for US government purposes. DOE will provide public access to these results of federally sponsored research in accordance with the DOE Public Access Plan (https://www.energy.gov/doe-public-access-plan).
