# Supplementary material for: Influence of Magnetic Sublattice Ordering on Skyrmion Bubble Stability in 2D Magnet Fe5GeTe2
Source: ACS Nano. 2024 Jul 8;18(28):18246–56. doi: 10.1021/acsnano.4c00853 (PMC11256745; doi:10.1021/acsnano.4c00853)
Supplement: Supplementary file 1 — nn4c00853_si_001.pdf [file nn4c00853_si_001.pdf]

Supporting Information: Influence of Magnetic Sublattice Ordering on Skyrmion Bubble Stability in 2D Magnet Fe_5GeTe_2

Max T. Birch,[*,†,‡] Fehmi S. Yasin,[‡,¶] Kai Litzius,[†] Lukas Powalla,[§] Sebastian Wintz,[‖] Frank Schulz,[†] Alexander E. Kossak,[⊥] Markus Weigand,[‖] Tanja Scholz,[§] Bettina V. Lotsch,[§,#] Gisela Schütz,[†] Xiuzhen Z. Yu,[‡] and Marko Burghard[*,§]

†*Max Planck Institute for Intelligent Systems, Heisenbergstraße 3, 70569 Stuttgart, Germany*

‡*RIKEN Center for Emergent Matter Science, Hirosawa 2-1, 351-0198 Wako, Japan*

¶*Center for Nanophase Materials Sciences, Oak Ridge National Laboratory, TN 37830, United States*

§*Max Planck Institute for Solid State Research, Heisenbergstraße 1, 70569 Stuttgart, Germany*

‖*Helmholtz-Zentrum Berlin für Materialien und Energie GmbH, Hahn-Meitner-Platz 1, Berlin, 14109 Germany*

⊥*Department of Materials Science and Engineering, Massachusetts Institute of Technology, Cambridge, MA 02139, USA.*

#*University of Munich (LMU), Butenandtstraße 5-13 (Haus D), 81377 München, Germany*

E-mail: maximilian.birch@riken.jp; m.burghard@fkf.mpg.de

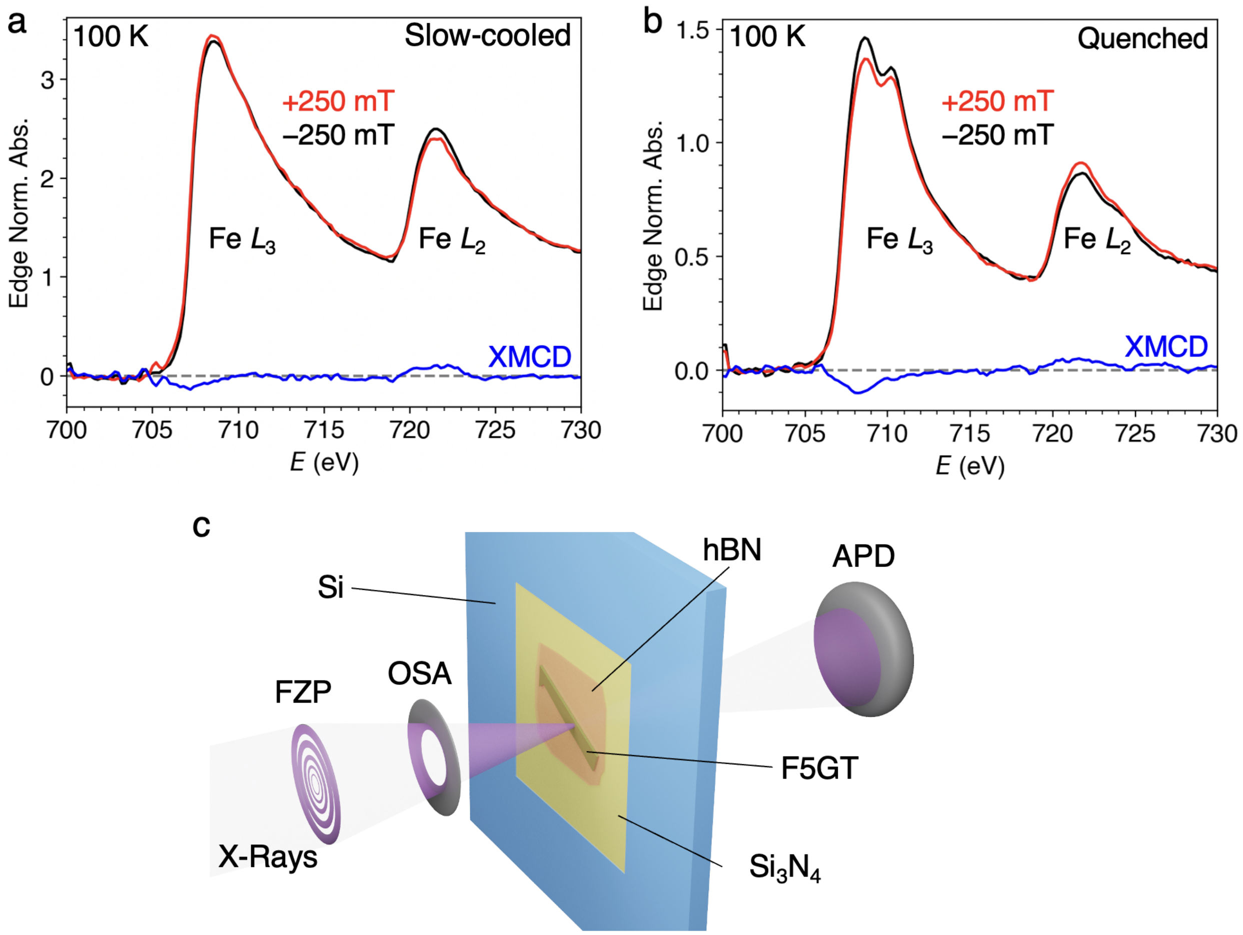

Supplementary Fig. S1: a,b) X-ray absorption spectra of the slow-cooled and quenched Fe_5GeTe_2 flakes, measured in transmission. Spectra measured under an applied field of ±250 mT (red/black) are plotted, as well as the corresponding x-ray magnetic circular dichroism (XMCD, blue) signal. The larger secondary absorption peak of the quenched flake may indicate higher relative thickness of the oxidized F5GT layers. c) Schematic illustration of the STXM measurement and sample construction. The Fresnel zone plate (FZP), order selecting aperture (OSA) and avalanche photodiode (APD) are labeled.

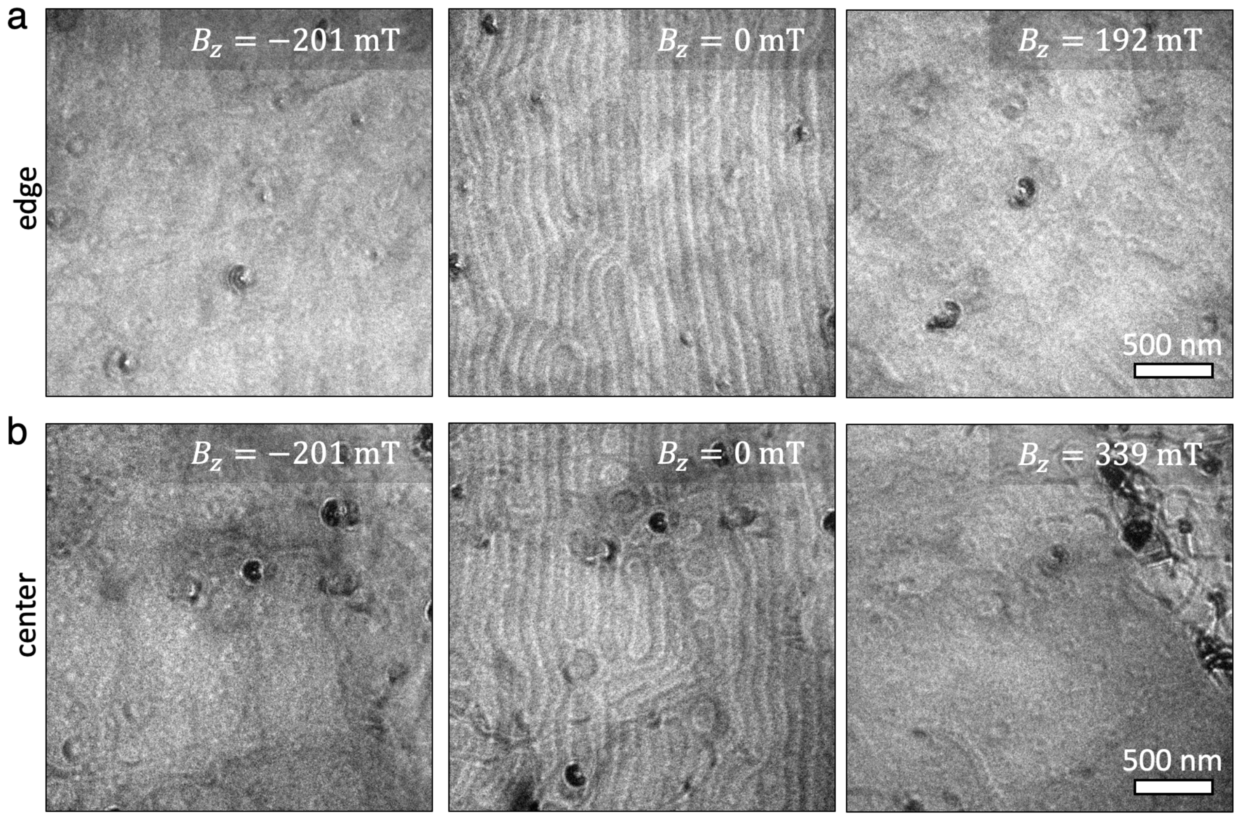

Supplementary Fig. S2: a,b) Example Lorentz transmission electron microscopy images at 250 K, measured at the two regions of interest in slow-cooled F5GT flake 2: the edge and the center, respectively.

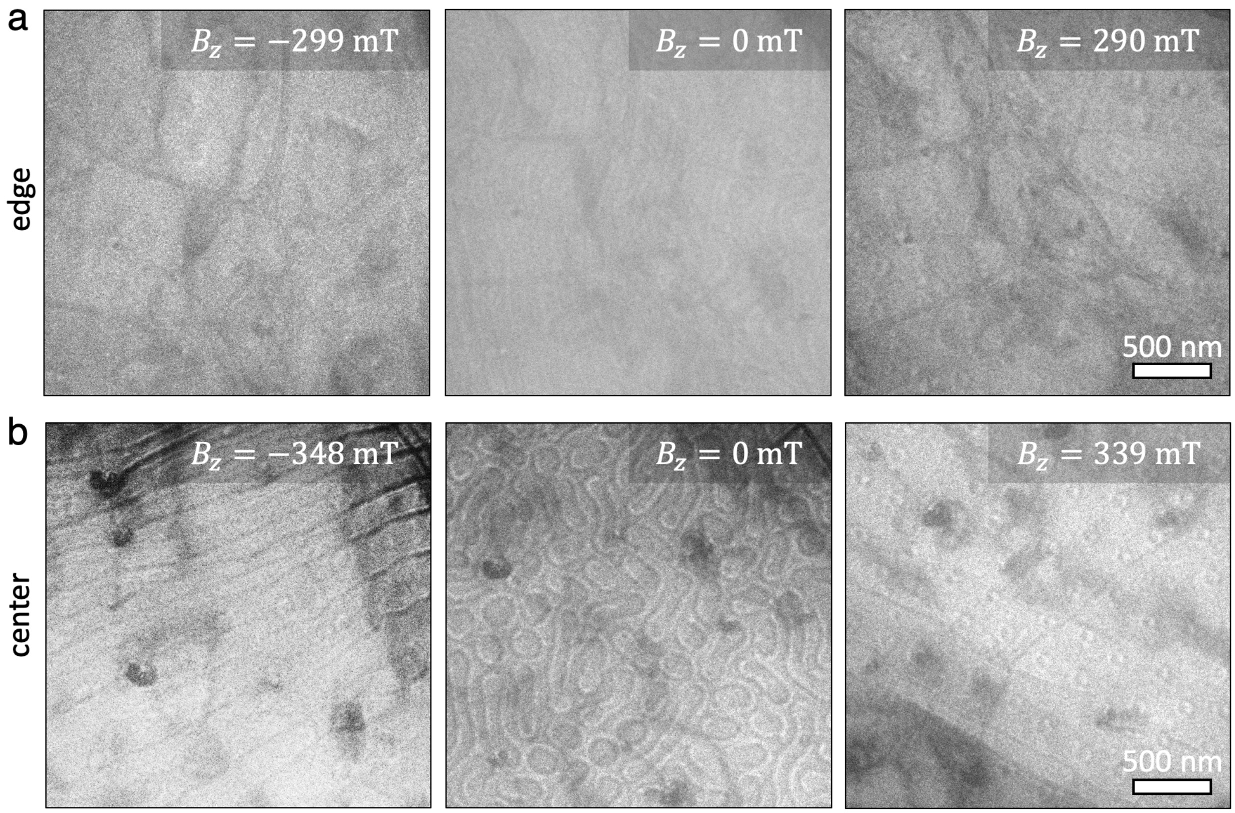

Supplementary Fig. S3: a,b) Example Lorentz transmission electron microscopy images at 200 K, measured at the two regions of interest in slow-cooled F5GT flake 2: the edge and the center, respectively.

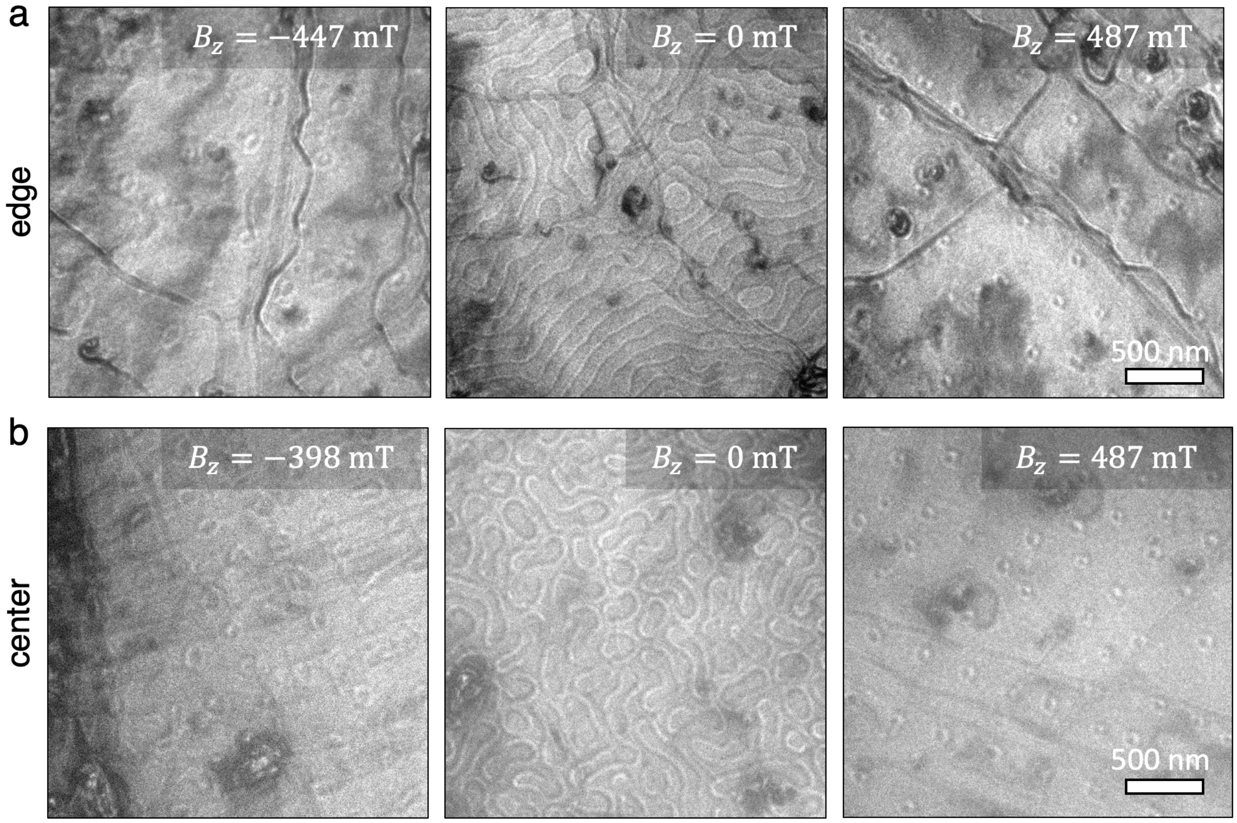

Supplementary Fig. S4: a,b) Example Lorentz transmission electron microscopy images at 98 K, measured at the two regions of interest in slow-cooled F5GT flake 2: the edge and the center, respectively.

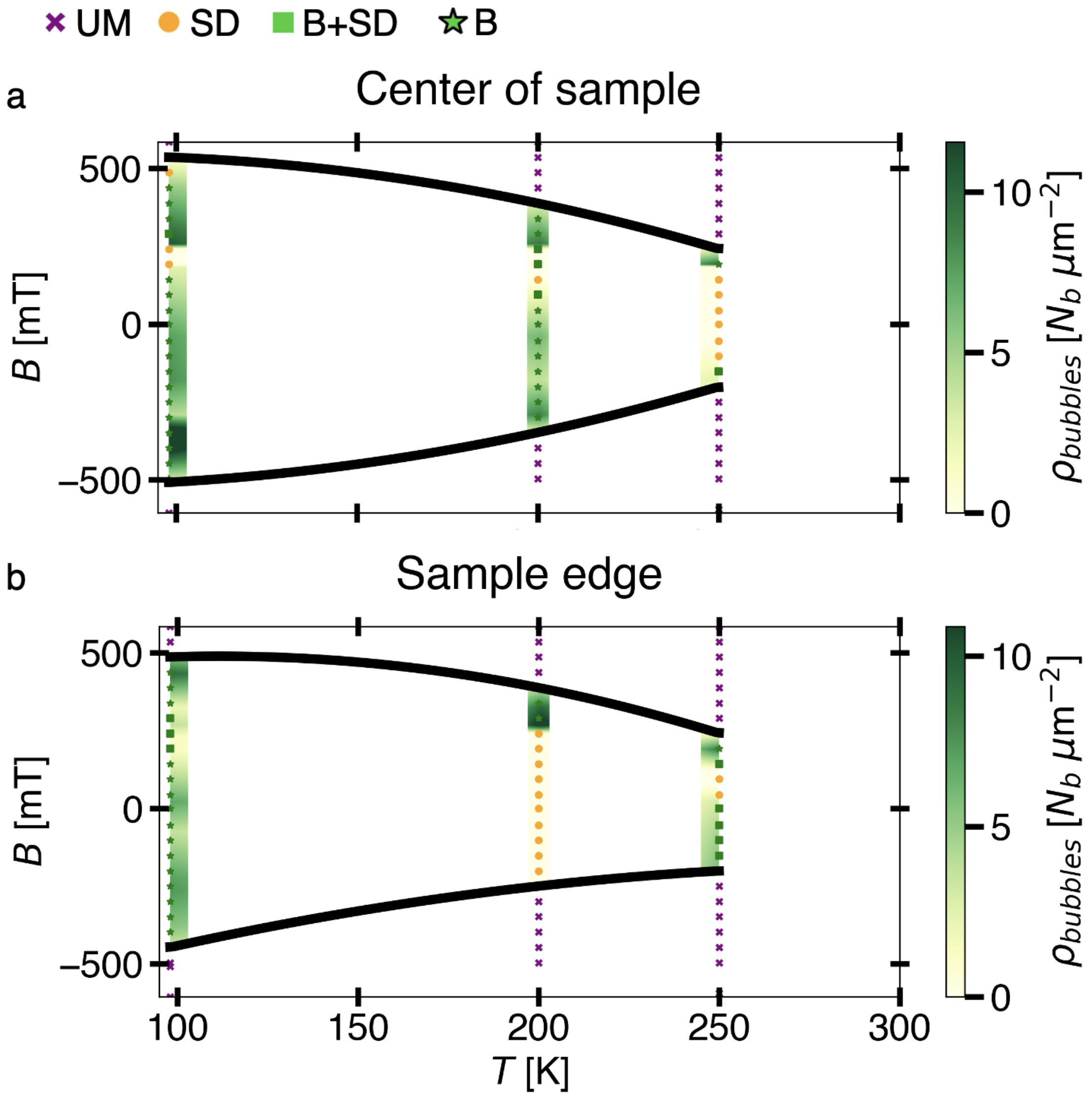

Supplementary Fig. S5: a,b) Magnetic phase diagrams of a few temperatures, as measured on slow-cooled Fe_5GeTe_2 flake 2 in the LTEM measurements, for the center and edge of the sample, respectively. The density of the bubbles is shown as a color map.

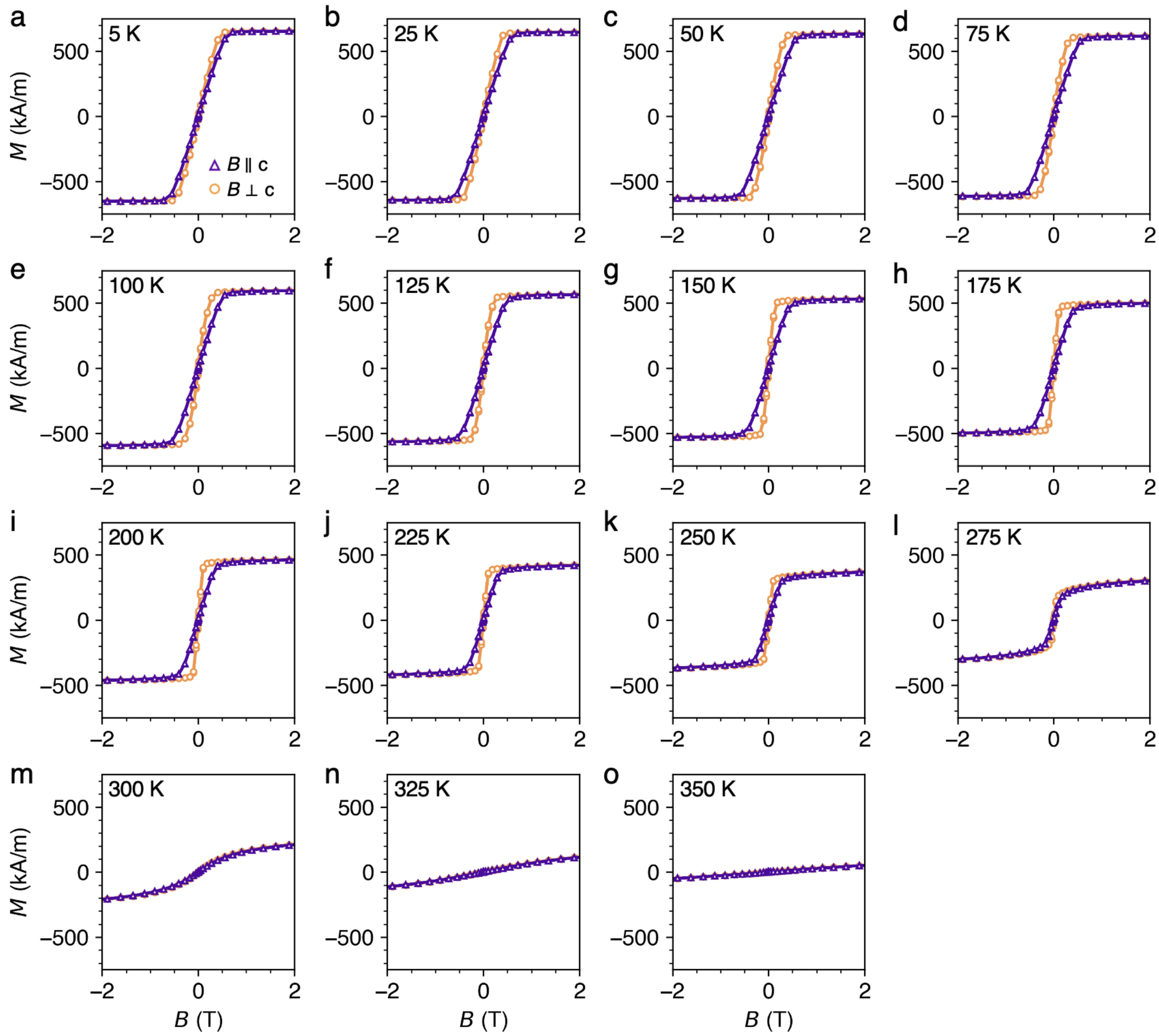

Supplementary Fig. S6: a-o) Magnetization M measured as a function of applied magnetic field B for the slow-cooled Fe_5GeTe_2 bulk single crystal. Data was acquired at a range of temperatures, with the field applied either out-of-plane along the c axis ($B \parallel c$), or in the ab plane ($B \perp c$). The data was utilized to calculate the estimated uniaxial anisotropy.

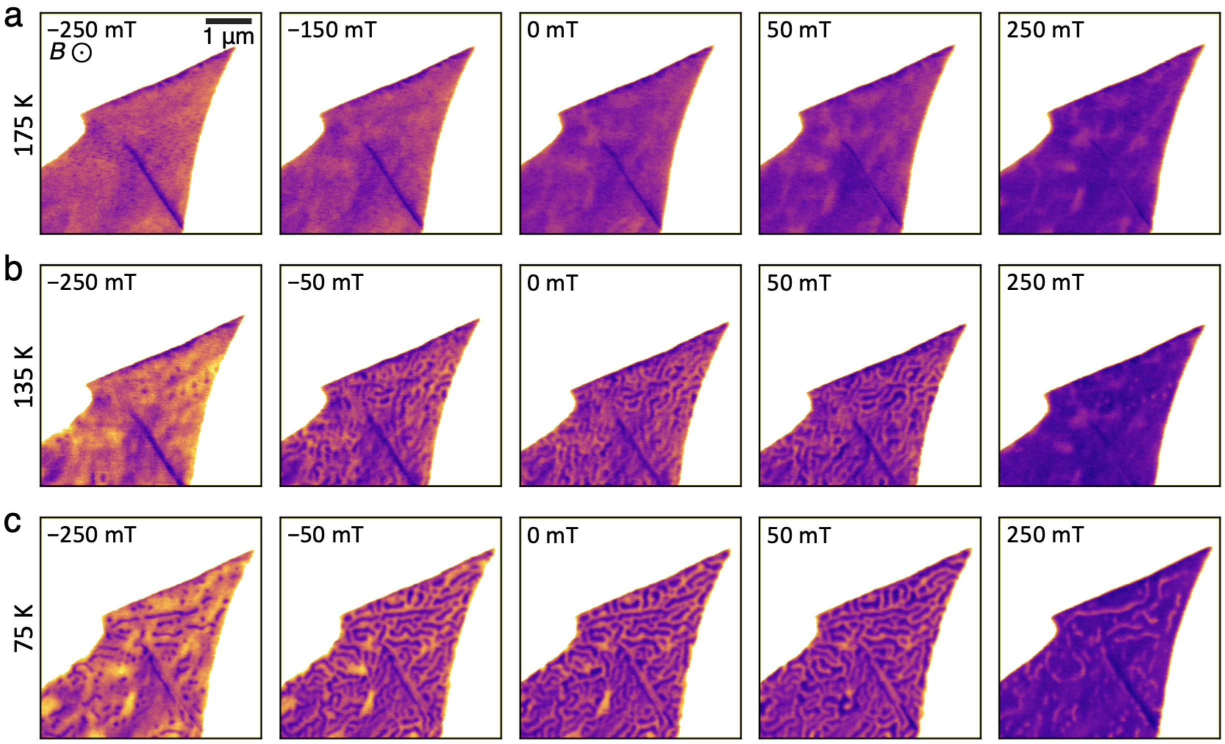

Supplementary Fig. S7: a-c) X-ray micrographs of another quenched Fe_5GeTe_2 flake, at selected temperatures and applied out-of-plane magnetic fields. The images are of a single x-ray polarisation, and therefore show both structural and magnetic contrast.

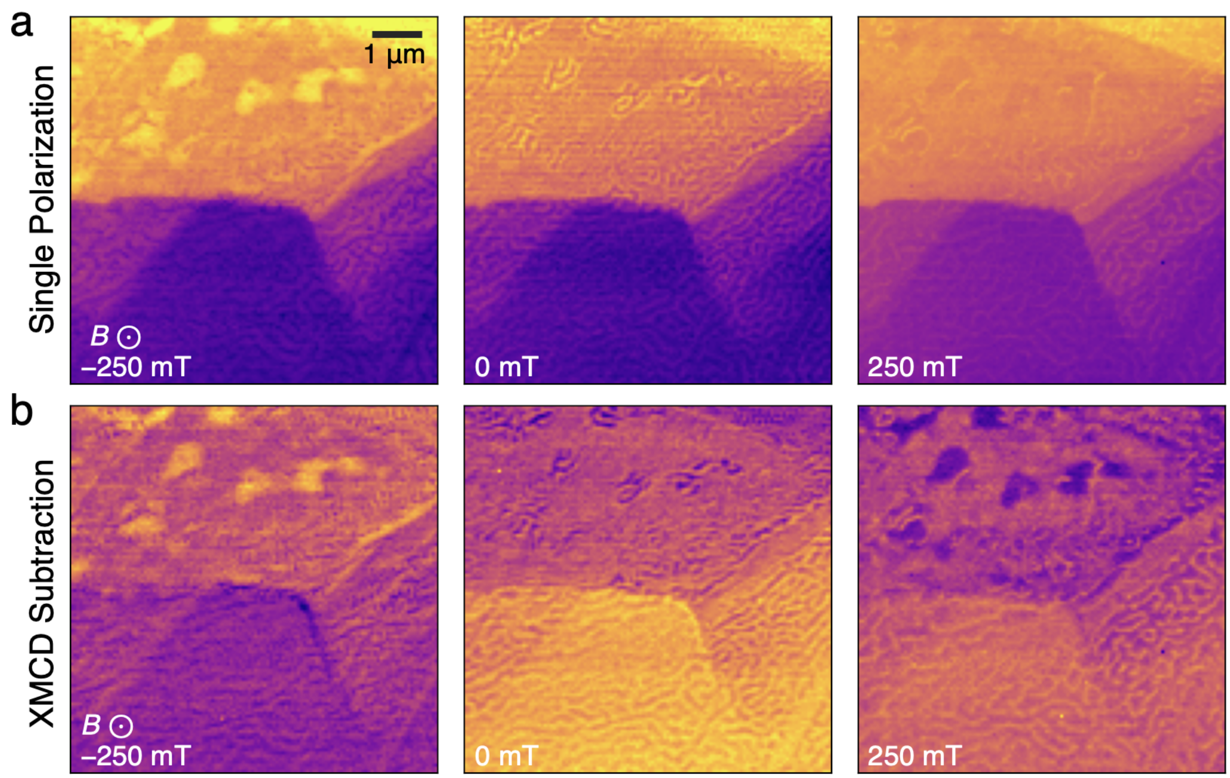

Supplementary Fig. S8: a,b) X-ray micrographs of another quenched Fe_5GeTe_2 flake, at applied out-of-plane magnetic fields, acquired at 80 K. The panels in a are of a single x-ray polarisation, and therefore show both structural and magnetic contrast. The panels in b display a subtraction of left and right circularly polarised x-ray images, and therefore show only the out-of-plane magnetic contrast.

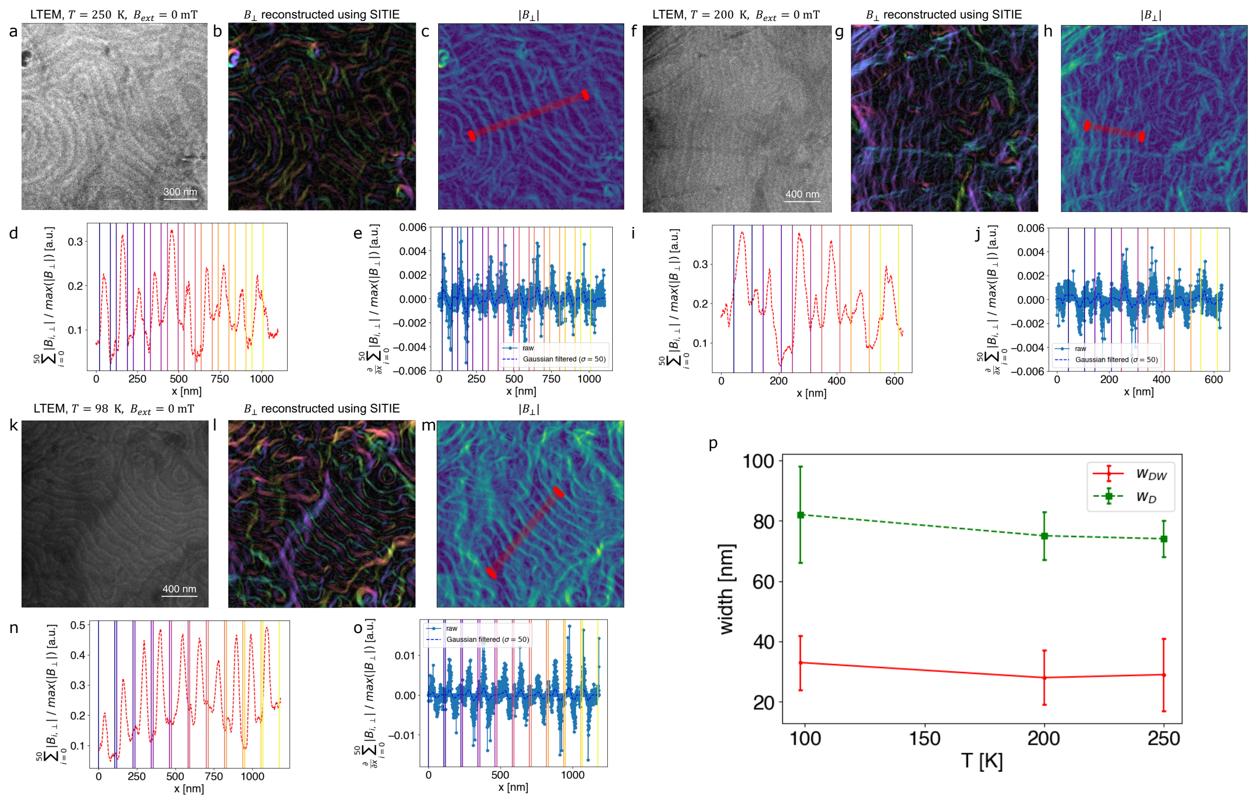

Supplementary Fig. S9: a, f, k) Zero-field LTEM micrographs b, g, l) in-plane magnetic induction reconstructed using single image TIE and c, h, m) magnitude of the in-plane magnetic induction $|B_\perp|$ of the stripe domains at T = 250 K, T = 200 K, and T = 98 K, respectively. d, I, n) Line profile (averaged over 50 lines) of $|B_\perp|$ and e, j, o) its derivative $\partial/\partial x \sum_{(i=0)}^{50} |B_\perp|/(|B_\perp^{\max}|)$ across the stripe domains plotted with vertical line windows indicated by different colors (cold to warm from left to right) containing individual domain walls at T = 250 K, T = 200 K, and T = 98 K, respectively. p) Plots of the domain wall width $w_{\rm DW}$ and characteristic stripe domain width $w_{\rm D}$ as a function of temperature measured as the distance between neighboring local gradient maxima and minima, and the neighboring local minima and maxima, respectively. The error bars represent the standard deviation from the mean.

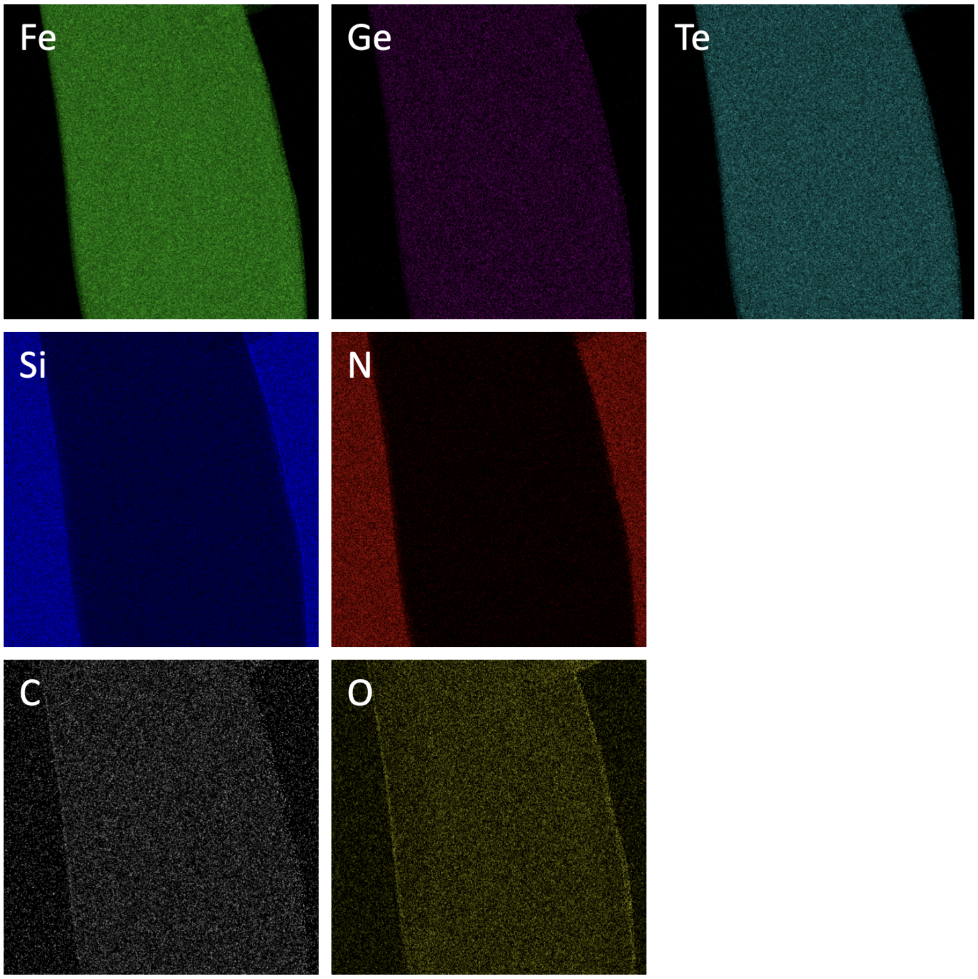

Supplementary Fig. S10: EDX measurements of slow-cooled Fe_5GeTe_2 flake 2, investigated in the LTEM experiments.
